# The Relationship of Neutrophil to Lymphocyte Ratio with Vitreomacular Traction Syndrome

**Published:** 2019

**Authors:** Cagri ILHAN, Mehmet CITIRIK, Mehmet Murat UZEL, Kemal TEKIN

**Affiliations:** 1 Department of Ophthalmology, Hatay State Hospital, Hatay, Turkey; 2 Department of Ophthalmology, University of Health Sciences, Ankara Ulucanlar Eye Education and Research Hospital, Ankara, Turkey; 3 Department of Ophthalmology, Afyonkarahisar State Hospital, Afyonkarahisar, Turkey; 4 Department of Ophthalmology, Kars Harakani State Hospital, Kars, Turkey

**Keywords:** Vitreomacular Traction Syndrome, Diabetic Vitreomacular Traction, İdiopathic Vitreomacular Traction, Neutrophil to Lymphocyte Ratio

## Abstract

This study was conducted to reveal the role of systemic low-grade inflammation by calculating the Neutrophil/Lymphocyte Ratio (NLR) in Vitreomacular Traction Syndrome (VMTS) of different etiologies. A retrospective examination was made of the medical records at a tertiary referral hospital. The study included 31 patients with diabetic VMTS, 27 patients with idiopathic VMTS, and 35 healthy subjects as the control group. The White Blood Cell (WBC) counts and Neutrophil-to-Lymphocyte Ratio (NLR) was compared. There were insignificant differences between the groups in terms of mean age and female/male ratio (P>0.05). The mean ± Standard Deviation (SD) of NLR was calculated as 2.43 ± 0.83 in the diabetic VMTS group, 2.38 0.96 in the idiopathic VMTS group, and 1.83 WBC: White blood cell; VMTS: Vitreomacular traction syndrome; SD: standard deviation; µL: microliter; fL: femtolitre; n: number. 0.52 in the control group (P=0.007). The values of the diabetic and idiopathic VMTS groups were significantly higher than those of the control group (P=0.002 and P=0.032, respectively). However, differences between the diabetic and idiopathic VMTS groups were insignificant (P=0.651). This study found significantly higher NLR in patients with diabetic and idiopathic VMTS than the control group. Elevated NLR could therefore be a potential indicator of VMTS, irrespective of the etiology.

## INTRODUCTION

Anomaly in Posterior Vitreous Detachment (PVD) has been considered as an significant factor in the pathogenesis of vitreomacular interface. The progression of PVD can cause traction on the macula. Traction on the retina can lead to anatomic alterations in the vitreomacular interface and subsequently results in loss of vision [1]. If the vitreous is separated from the peripheral retina yet remains adherent posteriorly and causes an anteroposterior traction region encompassing the macular area, then Vitreomacular Traction Syndrome (VMTS) occurs [2]. With the use of Optical Coherence Tomography (OCT), anteroposterior tractional force has been implicated as a reasonable cause of VMTS [3]. This traction can occur as idiopathic or may be related to retinal vascular diseases, posterior segment inflammatory diseases, trauma, surgery, retinal break, intraocular tumor, and miscellaneous ocular diseases. However, in diabetic retinopathy, VMTS can be a specific feature in presentation and management [4, 5]. The pigmented or non-pigmented cells of the ciliary body, retinal pigment epithelium cells, vascular system’s mesodermal elements, inflammatory or normal cells within the vitreous, or retinal glial cells consist of these tractional membranes and, stimulated in part by the inflammation. Those membranes are architecturally enhanced by the presence of fibrocytes and macrophages [6].

The evaluation of White Blood Cell (WBC) count, subtypes, and the calculation of their ratios to each other are useful markers of subclinical systemic inflammation [7]. The superiority of Neutrophil-to-Lymphocyte Ratio (NLR) to total leukocyte count has been shown in previous studies [8, 9]. The role of NLR in some systemic diseases has been reported, yet there has not yet been sufficient clarification of its relationship with ocular diseases [10-13]. 

To the best of the author’s knowledge, the association of NLR and VMTS has never been reported. This study aimed at revealing the role of systemic low-grade inflammation by calculating NLR in VMTS of different etiologies. 

## METHODS

This study was conducted by the ophthalmology department at a tertiary referral hospital. Approval for the study was decided by the institutional review board and ethics committee. All procedures were accomplished in line with the ethical standards of the Helsinki Declaration for human subjects. A retrospective evaluation was made of the medical records of patients, who underwent pars plana vitrectomy due to VMTS between 2013 and 2017. The preoperative records included medical history, ocular or systemic medication, detailed ocular examination and routine laboratory results, including WBC. Only patients with diabetic and idiopathic VMTS were meticulously selected and included in the study. Coexistence of additional ocular pathologies, such as retinal vascular diseases, retinal break, intraocular tumor, chronic ocular medication, the history of uveitis, retinal surgery, and ocular trauma are considered as exclusion criteria. Patients with hematological disorders, malignancy, acute or chronic infection, other inflammatory ocular and systemic diseases, a history of steroid use, or any ocular medication were also excluded. All diabetic patients with VMTS (diabetic VMTS group, n=31) had stages of diabetic retinopathy and other patients (idiopathic VMTS group, n=27) with no risk factor related to VMTS were included. The control group (n=35) was established from random selection of healthy subjects hospitalized for cataract surgery with no history of any ocular (except senile cataract) or systemic diseases (including diabetes mellitus). All the records of the participants, from which the information was extracted were coded, and without informing the patient's name, the required information was extracted and used for final analysis. Vitreomacular traction syndrome was diagnosed using a spectral domain OCT (Heidelberg Engineering, Heidelberg, Germany), according to the criteria of the OCT-based definition of VMTS by the International Vitreomacular Traction Study Group [2]. To confirm the VMT in an eye, all of the following anatomical criteria were observed in at least one B-mode OCT scan, which included: 1. existence of sign of perifoveal vitreous cortex detachment from the retinal surface; 2. macular attachment of the vitreous cortex in the range of three millimeters radius of the fovea; and 3. accompaniment attachment with distortion of the surface of the fovea, changes in the structure of the intra-retina, bulging of fovea above the retinal pigment epithelium, or a combination of them without interrupting the entire thickness of all retinal layers.

For all the study subjects evaluated with Horiba ABX Pentra 120 (Holliston, MA, USA), the neutrophil, lymphocyte, monocyte, platelet counts, and main platelet volume (MPV) values were retrieved from the hospital medical records. Neutrophil-to-lymphocyte, monocyte-to-lymphocyte, platelet-to-lymphocyte, and MPV-to-lymphocyte ratios were calculated by dividing the neutrophil, monocyte, platelet, and MPV counts by the lymphocyte count, respectively. 

Statistical analyses were performed using Statistical Package for the Social Sciences (SPSS) 24.0 software (IBM Corp, New York, USA). The mean age and female-to-male ratio of the groups were provided as descriptive data. The mean counts of WBC and calculations of defined variables were presented in tables. The fitting of numerical data to normal distribution was assessed by the Kolmogorov-Smirnov test. The non-parametric Kruskal-Wallis H test was applied to compare three independent samples when numerical data had not normal distribution. After Bonferroni correction, a P value of ≤0.05 was considered statistically significant. The Mann-Whitney U test was used for post-hoc analysis of two independent samples and a P value of ≤0.05 was considered statistically significant. Receiver Operating Characteristics Curve (ROC) analysis was performed to determine the sensitivity and specificity of admission of NLR and the optimal cut-off value for predicting VMTS (irrespective of the etiology of the VMTS).

## RESULTS

The mean and Standard Deviation (SD) for age of patients was 67.16 ± 7.07 years in the diabetic VMTS group, 71.24 ± 12.33 years in the idiopathic VMTS group, and 69.08 ± 8.42 years in the control group (P=0.253). The diabetic VMTS group comprised of 16 females and 15 males, the idiopathic VMTS group included 13 females and 14 males, and the control group had 17 females and 18 males (P = 0.727). 

**Figure 1:**
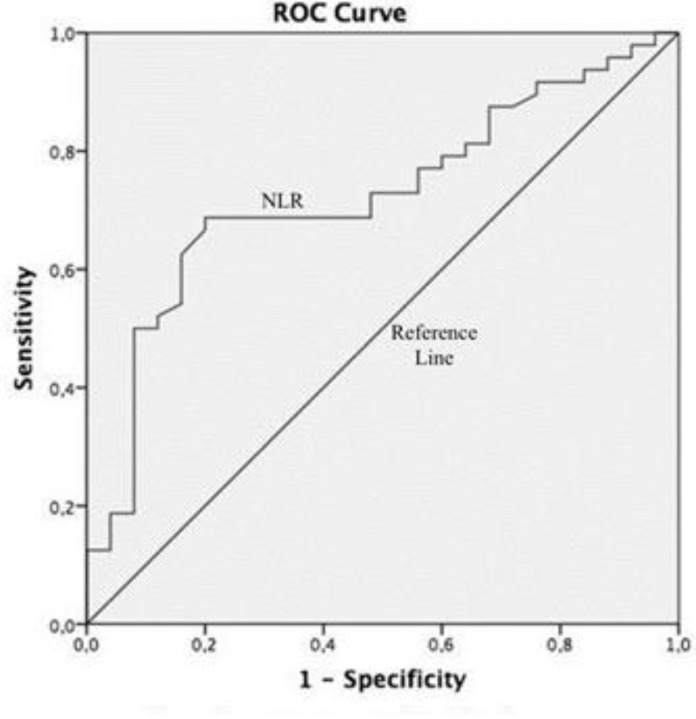
The area under the Receiver Operating Characteristics (ROC) Curve. In the ROC Analysis, the area under the curve for Neutrophil-to-Lymphocyte Ratio (NLR) was 0.724 and an NLR of 2.01 or higher predicted Vitreomacular Traction Syndrome (VMTS), İrrespective of the etiology with a sensitivity of 62% and specificity 84% (P-value= 0.02; 95% confidence interval 0.604 – 0.844).

The neutrophil, leukocyte, monocyte, platelet counts, and MPV values were similar in the three groups (all P values > 0.017). The mean±SD of Neutrophil-to-Lymphocyte Ratio was calculated as 2.43 ± 0.83 in diabetic VMTS, 2.38 ± 0.96 in idiopathic VMTS, and 1.83 ± 0.52 in the control group. There was a significant difference between the three groups regarding the NLR (P=0.007). In the post-hoc analysis, the values of the diabetic and idiopathic VMTS groups were significantly higher than those of the control group (P=0.002 in diabetic VMTS versus control, and P=0.032 in idiopathic VMTS versus control), yet there was insignificant difference between the diabetic and idiopathic VMTS groups (P = 0.651). The monocyte-to-lymphocyte, platelet-to-lymphocyte, and MPV-to-lymphocyte ratios were also similar in the three groups (P>0.017). The results of the comparisons of the mean WBC counts and the calculations of defined variables are shown in Table 1. In the ROC analysis, the area under the curve for NLR was 0.724, and an NLR of 2.01 or higher predicted VMTS (irrespective of the etiology of the VMTS) with a sensitivity of 62%, and specificity 84% (Fig 1).

**Table 1:** The comparison of the WBC counts and calculations of the defined variables of diabetic VMTS (n=31), idiopathic VMTS (n=27), and control (n=35) groups.

	Diabetic VMTS Mean ± SD	Idiopathic VMTS Mean ± SD	Control Mean ± SD	P value
Neutrophil count (x103L)	4.48 ± 1.46	3.95 ± 1.08	3.96 ± 1.00	0.407
Lymphocyte count (x103L)	1.94 ± 0.59	1.84 ± 0.76	2.26 ± 0.65	0.067
Monocyte count (x103L)	0.40 ± 0.33	0.31 ± 0.15	0.41 ± 0.18	0.157
Platelet count (x103L)	247.65 ± 89.55	232.41 ± 61.44	227.92 ± 50.70	0.533
Mean platelet volume (MPV)(fL)	8.23 ± 1.16	8.07 ± 1.30	8.01 ± 0.89	0.751
Neutrophil-to-lymphocyte ratio	2.43 ± 0.83	2.38 ± 0.96	1.83 ± 0.52	0.007*
Monocyte-to-lymphocyte ratio	0.20 ± 0.19	0.18 ± 0.08	0.23 ± 0.14	0.138
Platelet-to-lymphocyte ratio	122.35 ± 46.29	144.88 ± 68.85	128.48 ± 46.11	0.593
Main platelet volume-to-lymphocyte ratio	4.27 ± 1.37	5.04 ± 1.99	4.77 ± 2.47	0.318

WBC: White blood cell; VMTS: Vitreomacular traction syndrome; SD: standard deviation; µL: microliter; fL: femtolitre; n: number.

* P=0.002 in diabetic VMTS vs. control, and P=0.032 in idiopathic VMTS vs. Control. P values less than 0.05.

## Discussion

Aging is the main trigger of vitreous degeneration and PVD is the inevitable result of this condition in humans. The vitreous body shrinks with syneresis and the posterior hyaloid membrane becomes separated from the internal limiting membrane of retina. Posterior vitreous detachment consists of a number of phases occurring in a specific order. This physiological process can be affected negatively by several disorders, such as inflammation, trauma, and tumor, which result in the formation of anteroposterior and tangential contractile forces. Through different etiologies, astrocytes, myofibroblasts, and fibrocytes are known to play a key role in the occurrence of contraction in VMTS, yet the primary triggering mechanism of this process remains unclear [14, 15]. Takahashi et al. [16] revealed that the levels of numerous inflammatory cytokines are affected by the presence of PVD. These findings suggest that for various reasons, low-grade inflammation can cause VMTS.

Recent studies have emphasized that NLR is the new indicator of inflammatory activity related to several ocular diseases. Kurtul et al. [17] reported NLR to be a simple, inexpensive, and reliable prognostic biomarker of age-related macular degeneration. Ozgonul et al. [18] stated that NLR can be used as a novel biomarker in primary open-angle glaucoma and Li et al. [19] reported the diagnostic value of WBC counts in patients with primary angle-closure glaucoma. Dursun et al. [20] suggested that the optimal cut-off value of NLR was 1.89 with 72.5% sensitivity and 100% specificity to predict retinal vein occlusion. These reports revealed the diagnostic value of NLR and the critical roles of inflammatory cascades in the pathophysiology of these diseases. In the current study, it was found that a higher value of NLR than the cut-off value has prognostic importance for diabetic and idiopathic VMTS.

Many epidemiological studies have reported that diabetes mellitus is associated with chronic inflammation [21, 22]. According to a study by Ulu et al., [23] NLR is a quick and reliable predictor of the severity of the diabetic retinopathy. They found a significantly higher NLR in patients with diabetic retinopathy compared with those without diabetic retinopathy [23]. The results of the current study supported the mentioned study in the sense that NLR was significantly higher in patients with diabetic VMTS, consistent with the inflammatory pathogenesis. Moreover, NLR was also determined to be higher in idiopathic VMTS. 

Currently, the pathogenesis of idiopathic VMTS is not completely clear. Low-grade inflammation can be considered to trigger the presence of VMTS, independent of whether the patient is diabetic. 

The spontaneous detachment of vitreomacular traction depends on the dimensions of adhesion, the width of the vitreomacular angle, the treatment of simultaneous disease of the retina with intravitreal injections, and the vitreomacular interface area values [24-26]. Another important treatment in VMTS is the injection of ocriplasmin [27]. However, if both patient observation and medical treatment are unsuccessful or undesirable, pars plana vitrectomy is currently considered as the main treatment of the idiopathic VMTS [28]. There is ongoing research on the potential use of anti-inflammatory drugs in the treatment of several retinal diseases, such as age-related macular degeneration [29]. Understanding the pathogenesis of idiopathic VMTS brings new perspectives to treatment of the disease. It can be speculated that the development of medical anti-inflammatory treatment for idiopathic VMTS could replace pars plana vitrectomy.

To the best of the author’s knowledge, this is the first study investigating the relationship between NLR and VMTS. There were a number of limitations in this study. First, the sample size of the study was relatively small. Second, the data were not multicenter and the study design was retrospective case-control. Third, this study was not suitable for assessment of other potentially relevant factors, because the controls were healthy and did not have any systemic or ocular disease except cataract. 

## CONCLUSION

The results of this study demonstrate that NLR was significantly higher in diabetic and idiopathic VMTS than in healthy subjects. Elevated NLR can therefore be considered a potential indicator of VMTS, irrespective of the etiology. These results suggest that systemic low-grade inflammation plays a role in the development of diabetic or idiopathic VMTS. 
